# Diagnostic Value of lncRNAs as Biomarker in Hepatocellular Carcinoma: An Updated Meta-Analysis

**DOI:** 10.1155/2018/8410195

**Published:** 2018-10-15

**Authors:** Shilian Chen, Yaqin Zhang, Xuan Wu, Chaoyang Zhang, Guancheng Li

**Affiliations:** ^1^Key Laboratory of Carcinogenesis of the Chinese Ministry of Health and the Key Laboratory of Carcinogenesis and Cancer Invasion of Chinese Ministry of Education, Xiangya Hospital, Central South University, Changsha, China; ^2^Cancer Research Institute, Central South University, Changsha, China

## Abstract

Some long noncoding RNAs (lncRNAs) display aberrantly high or low expression in hepatocellular carcinoma (HCC) and have the potential to serve as diagnostic biomarkers. Here, we accomplished a meta-analysis based on current studies to assess the diagnostic value of lncRNAs in HCC. Eligible literatures were systematically selected from PubMed, Web of Science, and Embase (up to January 20, 2018) according to defined inclusion and exclusion criteria. QUADAS scale was applied to the quality assessment of the included studies. Statistical analysis was performed through bivariate random-effects models based on R software. Publication bias was evaluated by funnel plot and Begg's and Egger's tests. 16 articles containing 2,268 cancer patients and 2,574 controls were selected for the final meta-analysis. Random effect model was used for the meta-analysis due to significant between-study heterogeneity. The pooled sensitivity, specificity, diagnostic odds ratio (DOR), positive likelihood ratio (PLR), and negative likelihood ratio (NLR) were 0.87(0.838-0.897), 0.829(0.794-0.86), 23.085(20.575-25.901), 4.533(4.239-4.847), and 0.176(0.166-0.186), respectively. Summary receiver operating characteristic curve (SROC) was conducted to estimate the diagnostic accuracy of lncRNAs in HCC with the area under curve (AUC) of 0.915. Subgroups analysis showed that lncRNA profiling, sample size, specimen types, and ethnicity might be the sources of heterogeneity. No publication bias existed according to funnel plot symmetry and Begg's (*P *= 0.187) and Egger's (*P* = 0.477) tests. In conclusion, lncRNAs can serve as potential diagnostic biomarkers of HCC with high sensitivity and specificity. In addition, lncRNAs panel from serum and plasma has a relatively high diagnostic value for HCC patients from Asia.

## 1. Introduction

Liver cancer is one of the common malignant tumors with high incidence and mortality, which is of high prevalence in men and is a serious threat to public health especially in developing countries [1]. According to a study of cancer epidemiology in 2016, there are 39230 estimated new cases and 27170 estimated deaths cases of liver cancer in America [2]. However, the estimated new liver cancer cases and deaths are 466100 and 422100, respectively, in China in 2015, showing that the incidence cases and deaths are increasing over the past several years because of population growth and aging, although both the incidence rate and mortality are decreasing [3]. The risk factors of liver cancer include HBV and HCV infection, consumption of food with aflatoxin contamination, smoking, obesity, type II diabetes, cirrhosis, and nonalcoholic fatty liver disease [4–6]. The five-year survival rate of HCC is still low, although new therapy methods have been continually developed [7]. No significant clinical symptoms at early stage of HCC resulting in most patients missing the best treatment period is more crucial. Therefore, it is necessary to find effective biomarkers for early diagnosis of liver cancer to improve survival rate.

LncRNA, a kind of noncoding RNA with length more than 200 nucleotides participates in the regulation of gene expression, and its abnormal expression is closely related to cancer occurrence and development [8, 9]. Latest study has demonstrated that lncRNA AFAP1-AS1 was found to be upregulated in HCC, lung cancer, and esophageal squamous cell carcinoma (ESCC), and its overexpression conferred proliferation, invasion, and metastasis ability to cancer cell during the progression of malignant tumors [10–12]. LncRNA can also be used as prognostic factor to predict prognosis. MALAT-1 with abnormally high expression that could be an effective prognostic factor for various human cancers, especially non-small cell lung cancer [13]. Overexpressed HOTAIR is an independent prognostic factor for predicting HCC recurrence in liver transplantation patients [14]. Alpha Fetal Protein (AFP) is a clinically common tumor biomarker for diagnosis of HCC, but the sensitivity and specificity of AFP are relatively poor on clinic [15, 16]. Furthermore, AFP is of less diagnosis value when the liver tumor size is less than three centimeters [17]. In recent years, some studies have found that lncRNAs with abnormal high or low expression in body fluids can also serve as a tumor biomarker for early diagnosis of cancer [18]. Multiple overexpressed lncRNAs, including RP11-160H22.5, XLOC-014172, LOC149086, UCA1, WRAP53, AF085935, uc003wbd, PVT1, uc002mbe.2, PANDAR, SPRY4-IT1, uc001ncr, AX800134, linc00152, and HULC, have been identified to be prospective diagnostic indicators for HCC [19–34]. However, the sensitivity and specificity of different lncRNAs on the early diagnosis of HCC patients have been controversial. Thus, we conduct this meta-analysis to summarize the overall diagnostic performance of abnormally expressed lncRNAs for HCC to provide a reliable basis for clinic.

## 2. Materials and Methods

### 2.1. Literature Retrieval Strategy

We performed a literature search on up-to-date biomedicine database including PubMed, Web of Science, and Embase on January 20, 2018. In this process, we mainly searched three key factors: “liver cancer”, “lncRNA”, and “diagnosis”. The detailed search strategy for PubMed is as follows: (“Liver Neoplasms”[Mesh] OR “Hepatic Neoplasms”[tiab] OR “Hepatic Neoplasm”[tiab] OR “Liver Neoplasm”[tiab] OR “Liver Cancer”[tiab] OR “Liver Cancers”[tiab] OR “Hepatocellular Cancer”[tiab] OR “Hepatocellular Cancers”[tiab] OR “Hepatic Cancer”[tiab] OR “Hepatic Cancers”[tiab] OR HCC[tiab] OR “Hepatocellular Carcinoma”[tiab] OR “Hepatocellular Carcinomas”[tiab]) AND (“RNA, Long Noncoding”[Mesh] OR lncRNA*∗*[tiab] OR “Long ncRNA”[tiab] OR “Long Non-Translated RNA”[tiab] OR “Long Non-Coding RNA”[tiab] OR “Long Non Coding RNA”[tiab] OR “Long Non Protein Coding RNA”[tiab] OR “Long Non-Protein-Coding RNA”[tiab] OR “Long Noncoding RNA”[tiab] OR lncRNA*∗*[tiab] OR lincRNA*∗*[tiab] OR “Long ncRNAs”[tiab]) AND (diagnose[tiab] OR diagnosis[tiab] OR diagnostic[tiab] OR screen*∗*[tiab] OR detect*∗*[tiab]).

### 2.2. Inclusion and Exclusion Criteria

Studies were incorporated for the following criteria: (a) evaluating the diagnostic value of abnormally expressed lncRNAs in HCC; (b) being prospective or retrospective case-control studies; (c) research objects being human; (d) study subjects being definitely diagnosed by histopathology; (e) offering sufficient data including sample size, sensitivity, and specificity. Studies were excluded for the following criteria: (a) being irrelevant to lncRNA; (b) not being a study on HCC; (c) not being diagnostic study; (d) not being a study on human; (e) letters, reviews, or meta-analysis; (f) non-English articles; (g) short of full text; (h) insufficient data of diagnosis.

### 2.3. Data Extraction and Quality Assessment

Articles were independently screened by two reviewers (Chaoyang Zhang and Shilian Chen). The descriptive and quantitative information about lncRNA diagnosis value were extracted. Data extracted from the articles included the following items: first author, year of publication, country, ethnicity, lncRNA type, specimen type, sample size, sensitivity, specificity, and the areas under the curve (AUC). The quality of all the included studies was evaluated by the Quality Assessment of Diagnostic Accuracy Studies (QUADAS) scale. Each item of QUADAS was answered with “yes (Y)”, “no (N)”, and “unclear (U)”. The answer of “Y” means meeting the standard, while “N” or “U” means the dissatisfaction and unknown from the article, respectively.

### 2.4. Statistical Analysis

The software R was used for the statistical analysis of diagnostic data. Firstly, the test of heterogeneity among the included studies was conducted using Cochran-Q test. A* P* value < 0.01 for Cochran-Q test suggested a significant heterogeneity; therefore the random effect model was chosen for the computation of pooled indexes [35, 36]. The pooled sensitivity, specificity, diagnostic odds ratio (DOR), positive likelihood ratio (PLR), and negative likelihood ratio (NLR) were calculated using a bivariate analysis [37, 38]. Summary receiver operating characteristic (SROC) curve and the area under curve (AUC) were used to assess the diagnostic value of lncRNAs in HCC. Subgroup analysis was applied to seek the potential sources of heterogeneity among the studies. Funnel plot and Begg's and Egger's tests were applied to detect the publication bias of all the studies. A* P* values less than 0.05 was considered statistically significant.

## 3. Results

### 3.1. Literature Selection

**Figure 1:**
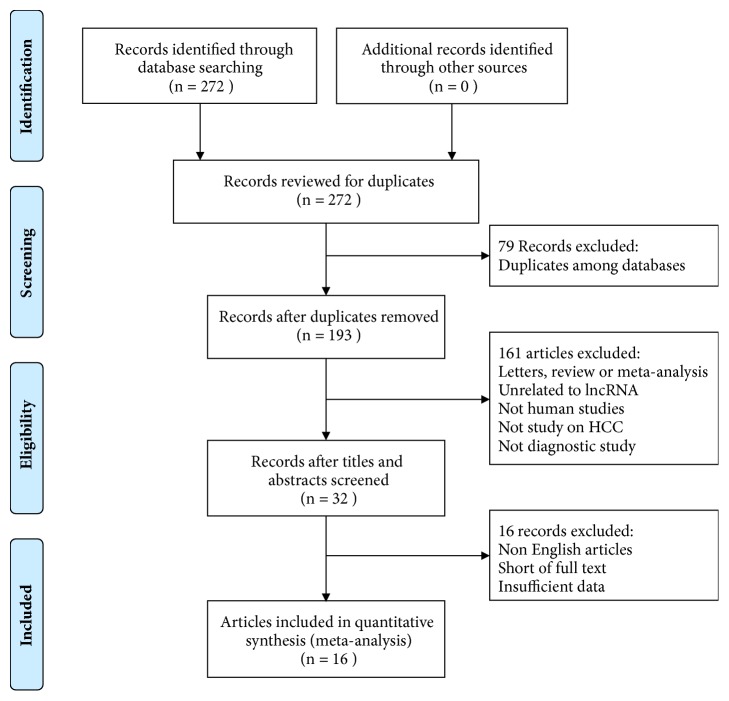
Selection process of articles included in the final meta-analysis.

A total of 272 records were identified from Embase, PubMed, and Web of Science, among which 79 articles were excluded due to duplication. After screening the titles and abstracts, 161 articles were excluded because they were letters, review articles, meta-analyses, unrelated to lncRNAs, not human studies, not study on HCC, and not diagnostic study. The remaining 32 records were used for further estimation, and 16 articles were excluded because they were non-English articles, short of full text, and short of sufficient data. Eventually, 16 eligible articles were included for the final meta-analysis. The flow diagram of the study selection was presented in Figure 1.

### 3.2. Study Characteristics and Quality Assessments

A total of 16 articles including 4842 samples were incorporated in the meta-analysis, involving 2268 cancer patients and 2574 controls. All the HCC patients had a definite diagnosis through the histopathological method. There were 24 kinds of lncRNAs derived from serum (n=10), plasma (n=12) and tissue (n=2), respectively. The quantitative reverse transcription polymerase chain reaction (qRT-PCR) was used for detecting lncRNA expression level. The primary clinical characteristics of the included studies were listed in Table 1. The quality assessment result for the studies according to 14 items of QUADAS checklist was shown in Table 2. As shown in the table, all the studies obtained QUADAS scores no less than 8, indicating a relatively high quality of the enrolled studies.

### 3.3. Diagnostic Performance

**Figure 2:**
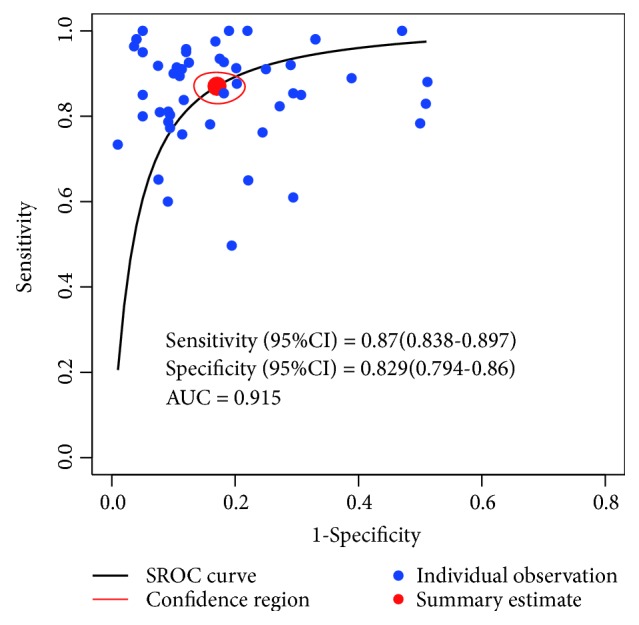
SROC curve for overall studies, AUC = 0.915.

The between-study heterogeneity was detected by Cochran-Q tests. Sensitivity (*P* < 0.01) and specificity (*P* < 0.01) indicated existing significant heterogeneity among all the studies. Therefore, the random effect model was selected for the meta-analysis. SROC curve for overall studies was displayed in Figure 2, in which lncRNA profiling showed a high diagnostic value (AUC = 0.915).

**Figure 3:**
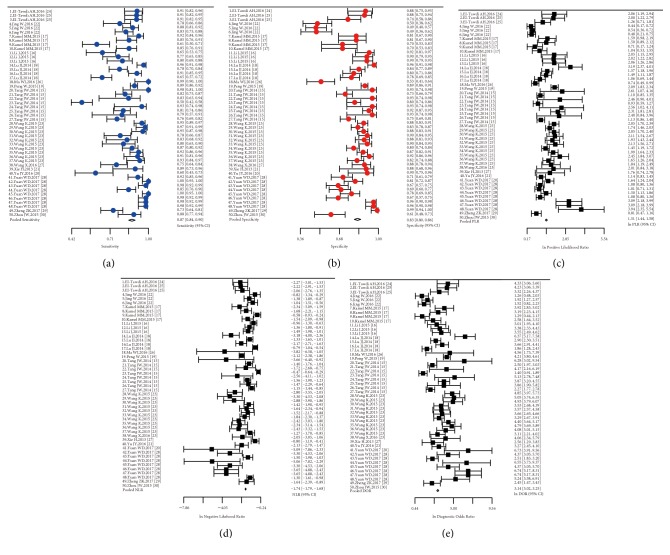
Forest plot. (a) The pooled sensitivity: 0.87(0.838-0.897); (b) the pooled specificity: 0.829(0.794-0.86); (c) the pooled lnPLR: 1.51(1.44-1.58); (d) the pooled lnNLR: -1.74(-1.79- -1.68); (e) the pooled lnDOR: 3.14(3.02-3.25).

Forest plots of the pooled sensitivity, specificity, DOR, PLR, and NLR for diagnostic performance of lncRNAs in HCC were shown in Figure 3. The pooled sensitivity, specificity, DOR, PLR, and NLR were 0.87(0.838-0.897), 0.829(0.794-0.86), 23.085(20.575-25.901), 4.533(4.239-4.847), and 0.176(0.166-0.186), respectively. The results indicated that lncRNAs with upregulated expression exhibited a relatively high diagnostic accuracy in HCC. More detailed results of meta-analysis existed in Table 3.

### 3.4. Subgroup Analysis

**Figure 4:**
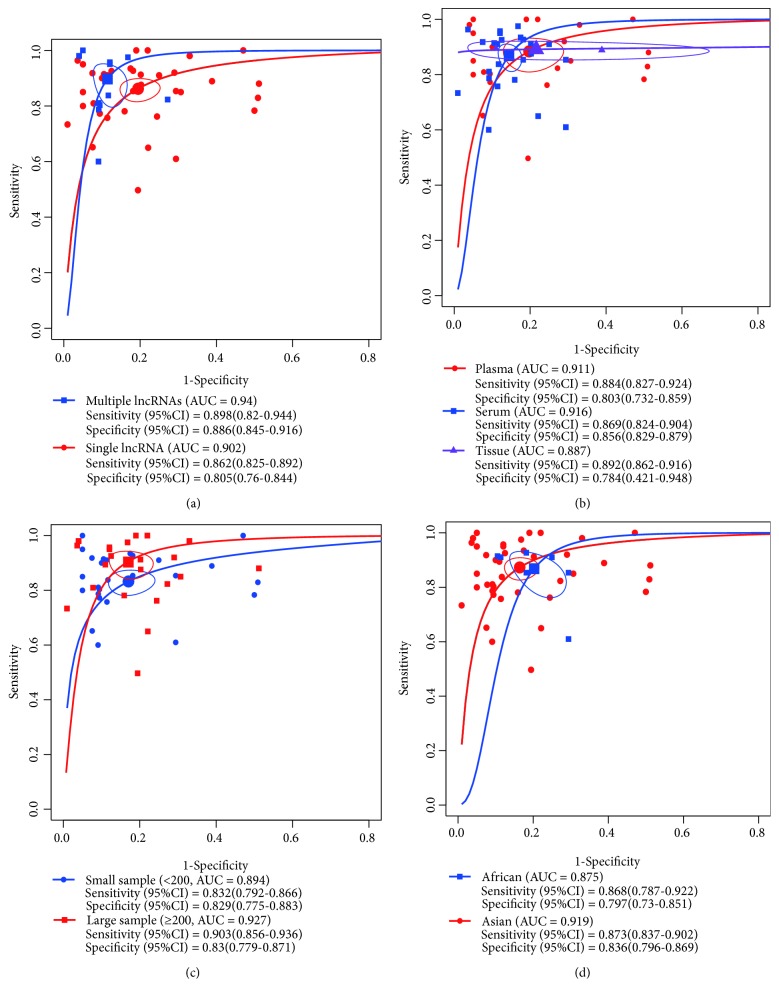
SROC curve for subgroup analysis. (a) SROC curve of single/multiple lncRNAs; (b) SROC curve of different sample source; (c) SROC curve of large/small sample size; (d) SROC curve of different ethnicity.

Stratified analyses were performed based on single or multiple lncRNAs, specimen types sample size, and ethnicity, which could seek potential sources of heterogeneity among studies. The SROC curve of subgroup studies were depicted in Figure 4. We first found that multiple lncRNAs achieved a higher accuracy than single lncRNA with sensitivity of 0.898 (0.82-0.944) versus 0.862 (0.825-0.892), specificity of 0.886 (0.845-0.916) versus 0.805 (0.76-0.844), and AUC of 0.94 versus 0.902, suggesting that existing an effective lncRNA panel such as RP11-160H22.5, XLOC_014172, and LOC149086, or Linc00152, RP11-160H22.5, and XLOC_014172, or HULC and Linc00152, or uc001ncr and AX800134, or PVT1 and uc002mbe.2 could perform combined diagnosis of HCC (Figure 4(a)). Secondly, we demonstrated that the diagnostic performance of lncRNA from serum and plasma was superior to lncRNA from tissue with sensitivity of 0.869 (0.824-0.904) and 0.884 (0.827-0.924) versus 0.892 (0.862-0.916), specificity of 0.856 (0.829-0.879) and 0.803 (0.732-0.859) versus 0.784 (0.421-0.948), and AUC of 0.916 and 0.911 versus 0.887, prompting that serum and plasma could be a better matrix for the diagnostic analysis of lncRNAs in HCC (Figure 4(b)). Then, subgroup analysis on sample size indicated that large sample (≥200) confirmed the high diagnostic performance of lncRNA in HCC compared with small sample (<200) with sensitivity of 0.903 (0.856-0.936) versus 0.832 (0.792-0.866), specificity of 0.83 (0.779-0.871) versus 0.829 (0.775-0.883), and AUC of 0.927 versus 0.894 (Figure 4(c)). Finally, We found that lncRNAs from Asian displayed higher diagnostic value than African with sensitivity of 0.873 (0.837-0.902) versus 0.868 (0.787-0.922), specificity of 0.836 (0.796-0.869) versus 0.797 (0.73-0.851), and AUC of 0.919 versus 0.875 (Figure 4(d)). Hence, these results suggested that heterogeneity among studies was mainly generated by lncRNA types, sample source, sample size, and ethnicity.

### 3.5. Publication Bias

**Figure 5:**
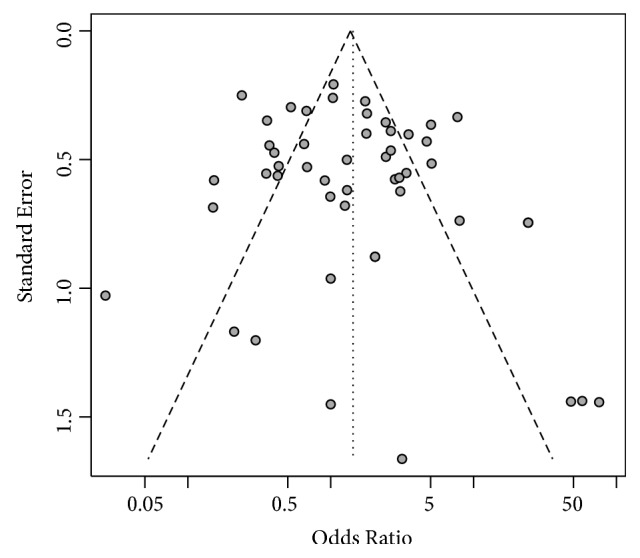
Funnel plot for publication bias; distribution of data points in funnel plot did not show apparent asymmetry.

Funnel plot and Begg's and Egger's tests were used to assess the possible publication bias of studies. As shown in Figure 5, distribution of data points in funnel plot did not show apparent asymmetry. In addition, Begg's rank correlation test and Egger's linear regression tests on funnel plot asymmetry further confirmed no significant publication bias with *P* values of 0.187 and 0.477, respectively. These results suggested that our meta-analysis results were stable and reliable.

## 4. Discussion

It is the first to evaluate the diagnostic value of lncRNAs in HCC base on R software. In the meta-analysis, there was significant heterogeneity existing in the finally incorporated studies, so we adopt the random effect model to further perform the meta-analysis. The pooled sensitivity, specificity, DOR, PLR, and NLR showed that lncRNAs have a high diagnostic value in HCC. Furthermore, by performing subgroup analysis to find the source of heterogeneity, we found that multiple lncRNAs from serum and plasma of Asian generated higher diagnostic value compared with single lncRNA from tissue of African. Meanwhile, Funnel plot and Begg's and Egger's tests demonstrated that no publication bias existed in the included studies. Consequently, we thought that lncRNA could be used as potential biomarker applied for early diagnosis of liver cancer in clinic.

We incorporated more articles about the diagnosis of HCC and provided more comprehensive assessment of the diagnostic performance of lncRNAs. The previous meta-analysis conducted by Hao et al. in 2017 included 19 studies from 10 articles with 1454 patients with HCC and 1300 controls [39], while our meta-analysis included 27 studies from 16 articles, containing more samples with 2268 cancer patients and 2574 controls. Therefore, this study not only reached conclusion consistent with Hao et al. study, but also more comprehensively and systematically evaluated the diagnostic performance of lncRNAs. lncRNAs as diagnostic biomarker for HCC were applicable to Asian population as well as African population. It is worth mentioning that the subgroup analysis of ethnicity in Hao et al. study showed African population had higher sensitivity and specificity than Asian population, whereas, after sample size was enlarged, our meta-analysis showed that the result was just the opposite. Despite this, we supported that the sample size was larger and the reliability was more accurate.

As almost ideally diagnostic biomarker of HCC, lncRNAs involved in our meta-analysis not only have high sensitivity and specificity of diagnosis, but also have high stability and long half-life period in general. There are, of course, also some other potential biomarkers for the diagnosis of HCC with the exception of lncRNA and AFP. Over the past several years, extensive researches have demonstrated that miRNAs are such a kind of biomarkers with relatively high sensitivity and specificity such as miRNA-375, miRNA-182, miR-21, miRNA-106b, and miRNA-183 [40–44]. In addition, some proteins specially expressed in HCC also have potential diagnostic value. Serum squamous cell carcinoma antigen (SCCA) has a moderate diagnostic value for HCC with pooled sensitivity of 0.61 and pooled specificity of 0.80 [45]. Osteopontin (OPN) shows a relatively high diagnostic accuracy for HCC with merged sensitivity of 0.86 and merged specificity of 0.86 [46]. Neuraminidase 1 (NEU1), which is upregulated in most HCC patients and promotes proliferation and migration, can serve as a novel biomarker for diagnosis in HCC with AUC of 0.87 [47]. In the meantime, emerging studies have found that epigenetics changes also have potential diagnostic value for various cancers including HCC. CpG loci of S100A8 methylation level are significantly decreased in HCC compared with the adjacent normal tissues, and S100A8 methylation can be served as potential diagnosis biomarker for HCC with a very high diagnostic accuracy (AUC=0.95) [48]. Consequently, combined diagnosis, in which diagnostic biomarkers include specially expressed AFP, miRNAs, lncRNAs, and DNA methylation, can acquire a higher diagnostic accuracy compared with traditional single detection.

At present, the most commonly used serum marker of HCC is AFP, because AFP expression level is related to HCC progression, the analytic method is simple, and the diagnostic standard is unified. However, using the AFP cutoff of 20ng/ml, the sensitivity and specificity by surface enhanced laser desorption/ionization time of flight mass spectrometry (SELDI-TOF MS) were 73% and 71%, respectively [49]. Interestingly, this study suggested lncRNAs showed higher diagnostic performance than AFP, where the sensitivity was 0.87 and the specificity was 0.829. Moreover, Zheng C. et al. [50] demonstrated that abnormal lncRNAs expression was associated with poor prognosis in HCC patients, indicating lncRNAs may be involved in the occurrence and development of disease, which provided favorable evidence for clinical application. In addition, serum lncRNAs showed higher sensitivity and specificity than plasma or tissue in this meta-analysis, so it is only necessary to collect blood sample from patients. The method for detecting lncRNAs expression level can be performed by quick and simple qRT-PCR, which is inexpensive. However, lncRNAs as a diagnostic biomarker for HCC is relatively less and lacks diagnostic criteria so that it limits its clinical application. In summary, when adding lncRNAs to AFP to diagnose HCC, which is a very cheap test, inevitably, this will be a crucial consideration from a cost-effectiveness perspective.

In our meta-analysis, there is also some insufficiency including small sample size, few lncRNA types, and only two diagnostic data from tissue. Furthermore, we did not conduct subgroup analysis on more clinical characteristics such as ages, gender, tumor stage, and lymphatic metastasis, which might be the source of between-study heterogeneity. Consequently, it needs more relevant studies and deeper data analysis to further confirm the overall diagnostic value of lncRNA in HCC.

## 5. Conclusions

By meta-analysis, we found that some abnormally expressed lncRNAs, especially multiple lncRNAs from serum and plasma, could be used as potential biomarker and had relatively high diagnostic accuracy in HCC. However, more studies need to be conducted to confirm the diagnostic value of lncRNA in HCC. Moreover, combined detection of different biomarkers could further improve the diagnostic performance in HCC.

## Figures and Tables

**Table 1:** Clinical characteristics of 27 studies included in meta-analysis.

**Author**	**Year**	**Ethnicity**	**LncRNAs**	**Sample types**	**SEN**	**SPE**	**Pat(n)**	**Con (n)**	**AUC**	**REF**
El-Tawdi AH	2016	African	UCA1	serum	0.914	0.886	70	38	0.91	[28]
El-Tawdi AH	2016	African	CTBP	serum	0.91	0.885	78	44	0.91	[29]
El-Tawdi AH	2016	African	CTBP	serum	0.91	0.75	78	36	0.83	[29]
Jing W	2016	Asian	SPRY4-IT1	plasma	0.783	0.5	60	63	0.702	[26]
Jing W	2016	Asian	GAS5	plasma	0.877	0.485	117	129	0.734	[26]
Jing W	2016	Asian	GAS5	plasma	0.833	0.491	117	55	0.688	[26]
Kamel MM	2016	African	UCA1	serum	0.927	0.821	82	44	0.861	[21]
Kamel MM	2015	African	WRAP53	serum	0.854	0.821	82	44	0.896	[21]
Kamel MM	2015	African	UCA1	serum	0.61	0.71	82	34	0.728	[21]
Kamel MM	2015	African	WRAP53	serum	0.854	0.71	82	34	0.787	[21]
Li J	2015	Asian	HULC	plasma	0.65	0.92	66	53	0.78	[20]
Li J	2015	Asian	Linc00152	plasma	0.768	0.902	66	53	0.85	[20]
Li J	2015	Asian	HULC, Linc00152	plasma	0.798	0.904	66	53	0.87	[20]
Lu JJ	2014	Asian	AF085935	serum	0.963	0.966	137	138	0.96	[22]
Lu JJ	2014	Asian	uc003wbd	serum	0.778	0.843	137	138	0.86	[22]
Lu JJ	2014	Asian	AF085935	serum	0.912	0.794	137	104	0.86	[22]
Lu JJ	2014	Asian	uc003wbd	serum	0.653	0.781	137	104	0.7	[22]
Ma WJ	2016	Asian	JPX	plasma	1	0.524	42	68	0.814	[30]
Peng W	2015	Asian	PANDAR	tissue	0.895	0.891	482	482	0.956	[23]
Tang JW	2015	Asian	RP11-160H22.5, XLOC_014172	plasma	1	0.95	20	20	1	[19]
			LOC149086							
Tang JW	2014	Asian	RP11-160H22.5, XLOC_014172	plasma	0.82	0.73	147	180	0.9	[19]
			LOC149086							
Tang JW	2014	Asian	RP11-160H22.5	plasma	0.85	0.953	20	20	0.9	[19]
Tang JW	2014	Asian	RP11-160H22.5	plasma	0.496	0.808	147	180	0.601	[19]
Tang JW	2014	Asian	XLOC_014172	plasma	0.95	0.95	20	20	0.95	[19]
Tang JW	2014	Asian	XLOC_014172	plasma	0.81	0.923	147	180	0.866	[19]
Tang JW	2014	Asian	LOC149086	plasma	0.8	0.953	20	20	0.875	[19]
Tang JW	2014	Asian	LOC149086	plasma	0.762	0.757	147	180	0.759	[19]
Wang K	2015	Asian	uc001ncr, AX800134	serum	0.95	0.881	121	232	0.949	[27]
Wang K	2015	Asian	uc001ncr, AX800134	serum	0.975	0.831	81	232	0.937	[27]
Wang K	2015	Asian	uc001ncr, AX800134	serum	0.957	0.881	70	232	0.945	[27]
Wang K	2015	Asian	uc001ncr, AX800134	serum	0.787	0.909	61	120	0.949	[27]
Wang K	2015	Asian	uc001ncr, AX800134	serum	0.846	0.884	37	120	0.953	[27]
Wang K	2015	Asian	uc001ncr, AX800134	serum	0.811	0.909	37	120	0.956	[27]
Wang K	2015	Asian	uc001ncr	serum	0.875	0.799	121	232	0.886	[27]
Wang K	2015	Asian	uc001ncr	serum	0.927	0.877	121	232	0.925	[27]
Wang K	2015	Asian	AX800134	serum	0.918	0.926	61	120	0.947	[27]
Wang K	2015	Asian	AX800134	serum	0.934	0.821	61	120	0.888	[27]
Wang X	2016	Asian	LINC01225	serum	0.761	0.88	66	70	0.886	[31]
Xie H	2013	Asian	HULC	plasma	0.9	0.9	30	20	0.86	[25]
Yu JY	2016	Asian	PVT1, uc002mbe.2	serum	0.601	0.901	40	33	0.764	[24]
Yuan WD	2017	Asian	Linc00152	plasma	0.92	0.709	100	100	0.869	[32]
Yuan WD	2017	Asian	RP11-160H22.5	plasma	1	0.811	100	100	0.884	[32]
Yuan WD	2017	Asian	XLOC_014172	plasma	0.978	0.668	100	100	0.759	[32]
Yuan WD	2017	Asian	Linc00152	plasma	0.846	0.695	100	100	0.826	[32]
Yuan WD	2017	Asian	RP11-160H22.5	plasma	1	0.778	100	100	0.859	[32]
Yuan WD	2017	Asian	XLOC_014172	plasma	0.981	0.673	100	100	0.735	[32]
Yuan WD	2017	Asian	Linc00152, RP11-160H22.5	plasma	0.978	0.957	100	100	0.986	[32]
			XLOC_014172							
Yuan WD	2017	Asian	Linc00152, RP11-160H22.5	plasma	0.981	0.961	100	100	0.985	[32]
			XLOC_014172							
Zheng ZK	2017	Asian	UCA1	serum	0.733	0.99	105	105	0.902	[33]
Zhou JW	2015	Asian	KLF4-003	tissue	0.889	0.611	54	54	0.803	[34]

SEN: sensitivity; SPE: specificity; Pat: patient; Con: control; AUC: area under the curve; REF: reference.

**Table 2:** QUADAS assessment for the studies included in meta-analysis for diagnosis.

**Fist author**	**Iterm 1**	**Iterm 2**	**Iterm 3**	**Iterm 4**	**Iterm 5**	**Iterm 6**	**Iterm 7**	**Iterm 8**	**Iterm 9**	**Iterm 10**	**Iterm 11**	**Iterm 12**	**Iterm 13**	**Iterm 14**	**Q**	**References**
Tang JW	Y	U	Y	U	Y	Y	Y	Y	U	U	Y	Y	N	Y	9	[19]
Li J	Y	Y	Y	U	Y	Y	Y	Y	U	U	Y	Y	N	Y	10	[20]
Kamel MM	Y	U	Y	U	Y	Y	Y	Y	U	U	Y	Y	N	Y	9	[21]
Lu JJ	Y	Y	Y	U	Y	Y	Y	Y	U	U	Y	Y	U	Y	10	[22]
Peng W	Y	U	Y	U	Y	Y	Y	Y	U	U	Y	Y	N	Y	9	[23]
Yu JY	Y	U	Y	U	Y	Y	Y	Y	U	U	Y	Y	N	Y	9	[24]
Xie H	Y	U	Y	U	Y	Y	Y	Y	U	U	Y	U	U	Y	8	[25]
Jing W	Y	U	Y	U	Y	Y	Y	Y	U	U	Y	Y	N	Y	9	[26]
Wang K	Y	Y	Y	U	Y	Y	Y	Y	U	U	Y	Y	N	Y	10	[27]
El-Tawdi AH	Y	U	Y	U	Y	Y	Y	Y	U	U	Y	Y	N	Y	9	[28]
El-Tawdi AH	Y	U	Y	N	Y	Y	Y	Y	U	U	Y	Y	N	Y	9	[29]
Ma WJ	Y	Y	Y	U	Y	Y	Y	Y	U	U	Y	Y	N	Y	10	[30]
Wang X	Y	U	Y	U	Y	Y	Y	Y	U	U	Y	Y	U	Y	9	[31]
Yuan WD	Y	U	Y	Y	Y	Y	Y	Y	U	U	Y	Y	N	Y	10	[32]
Zheng ZK	Y	Y	Y	U	Y	Y	Y	Y	U	U	Y	Y	N	Y	10	[33]
Zhou JW	Y	U	Y	N	Y	Y	Y	Y	U	U	Y	Y	N	Y	9	[34]

**Table 3:** Summarized results of meta-analysis based on R.

**Subgroup analysis**	**SEN(95**%**CI)**	**SPE(95**%**CI)**	**DOR(95**%**CI)**	**PLR(95**%**CI)**	**NLR(95**%**CI)**	**AUC**
**Hepatocellular carcinoma**	0.87(0.838-0.897)	0.829(0.794-0.86)	23.085(20.575-25.901)	4.533(4.239-4.847)	0.176(0.166-0.186)	0.915
**LncRNA profiling**						
Single lncRNA	0.862(0.825-0.892)	0.805(0.76-0.844)	20.027(17.638-22.740)	4.129(3.839-4.441)	0.191(0.179-0.204)	0.902
Multiple lncRNAs	0.898(0.82-0.944)	0.886(0.845-0.916)	43.220(32.833-56.893)	6.736(5.627-8.064)	0.127(0.115-0.140)	0.940
**Sample source**						
Plasma	0.884(0.827-0.924)	0.803(0.732-0.859)	14.132(11.992-16.654)	3.449(3.118-3.815)	0.203(0.187-0.221)	0.911
Serum	0.869(0.824-0.904)	0.856(0.829-0.879)	35.971(30.089-43.002)	6.057(5.504-6.666)	0.160(0.147-0.173)	0.916
Tissue	0.892(0.862-0.916)	0.784(0.421-0.948)	52.000(35.881-75.359)	6.473(5.075-8.256)	0.123(0.099-0.153)	0.887
**Sample size**						
Small sample(<200)	0.832(0.792-0.866)	0.829(0.775-0.883)	18.825(15.341-23.101)	4.065(3.652-4.524)	0.211(0.189-0.236)	0.894
Large sample(*⩾*200)	0.903(0.856-0.936)	0.83(0.779-0.871)	25.207(21.927-28.979)	4.736(4.343-5.164)	0.162(0.152-0.173)	0.927
**Ethnicity**						
Asian	0.873(0.837-0.902)	0.836(0.796-0.869)	23.549(20.864-26.579)	4.575(4.256-4.917)	0.175(0.166-0.185)	0.919
African	0.868(0.787-0.922)	0.797(0.73-0.851)	21.042(14.488-30.561)	4.293(3.538-5.211)	0.178(0.124-0.255)	0.875

SEN, sensitivity; SPE, specificity; DOR, diagnostic odds ratio; PLR, positive likelihood ratio; NLR, negative likelihood ratio; AUC, area under the curve.
